# Congenital absence of appendix: a rare condition that could result in severe complications—a review of literature

**DOI:** 10.1093/jscr/rjad661

**Published:** 2023-12-14

**Authors:** Mohamed Said Ghali, Nitasha Saleem, Mohamed H Khalaf, Ismail K Alkubaisi, Abdulhameed Ali, Mohammed Al Obahi, Raed M Al-Zoubi

**Affiliations:** Department of Surgery, Acute Care Surgery, Hamad Medical Corporation, P. O. Box 3050, Doha, Qatar; Department of General Surgery, Ain Shams University, 11566, Cairo, Egypt; Department of Surgery, Acute Care Surgery, Hamad Medical Corporation, P. O. Box 3050, Doha, Qatar; Department of Surgery, Acute Care Surgery, Hamad Medical Corporation, P. O. Box 3050, Doha, Qatar; Department of Surgery, Acute Care Surgery, Hamad Medical Corporation, P. O. Box 3050, Doha, Qatar; Department of Surgery, Acute Care Surgery, Hamad Medical Corporation, P. O. Box 3050, Doha, Qatar; Department of Surgery, Acute Care Surgery, Hamad Medical Corporation, P. O. Box 3050, Doha, Qatar; College of Medicine, Qatar University, P. O. Box 2713, Doha, Qatar; Surgical Research Section, Department of Surgery, Hamad Medical Corporation, P. O. Box 3050, Doha, Qatar; Department of Biomedical Sciences, QU-Health, College of Health Sciences, Qatar University, P. O. Box 2713, Doha, Qatar

**Keywords:** appendix agenesis, acute appendicitis, surgery, hemicolectomy

## Abstract

Acute appendicitis is the most frequent cause of abdominal pain and acute emergency surgeries, with a mortality risk of 6–7% at its onset. Since atypical deviations in these structures are rare, they can lead to diagnosis confusion and increase the risk of a worsening of the patient’s clinical picture. We present the case of a 35-year-old patient who had surgery after being diagnosed with acute appendicitis. Based on clinical assessment (Alvarado score 8), appendix agenesis was discovered intraoperatively and confirmed by postoperative pathology. Excess dissection while looking for the appendix caused an intraoperative complication of cecal damage, which was treated with a right hemicolectomy. Until now, just a few cases have been described in the literature. We record this case owing to its rarity and with the goal of further understanding the illness, which will lead to improved surgical results in similar patients.

## Introduction

Agenesis, which refers to full or partial absence, is one of the congenital disorders of the vermiform appendix. Although rare, only a few cases have been recorded so far [1, 2]. Morgagni reported the first incidence of appendiceal agenesis in 1719. Appendicular agenesis is often diagnosed perioperatively. It occurs in 1 in every 100 000 laparotomies performed due to a suspicion of acute appendicitis [3]. Due to the rarity of the event, the likelihood of appendiceal agenesis remains a challenge for the surgeon intraoperatively when difficulties in identifying the appendix are encountered. We provide a case of a patient who was clinically diagnosed with acute appendicitis and was referred for surgery, which was complicated by iatrogenic cecum perforation while trying to locate the appendix. Later, after a laparotomy and post-op pathology, it was discovered that the appendix was missing.

## Case report

A previously healthy 35-year-old male patient arrived at the ED with right lower abdomen pain and vomiting for 3 days. According to the patient’s evidence, he was feeling relatively well 3 days ago when he began to have discomfort, which was initially periumbilical and subsequently migrated to the right iliac fossa. The pain began as dull and painful and steadily rose in intensity. There were no elements that were aggravating or alleviating. Pain was linked to several episodes of vomiting and nausea, with no reported fever. On general examination, his vital signs were stable. On examination of his abdomen, it was not distended; there was no evident mass or previous surgical scars. Tenderness and localized guarding were present on superficial palpation of the right lower abdomen. Rovsing’s sign and rebound tenderness were present over the right iliac fossa. No organomegaly was found. On auscultation, bowel sounds were present. Other systemic examinations were within the normal limit. Laboratory parameters revealed neutrophilic leukocytosis with a total leucocyte count of 11.9 mcL and a neutrophil count of 82.2%. Other biochemical parameters were reasonably normal. A provisional diagnosis of acute appendicitis was made based on the Alvarado Scoring System, as the patient had a score of 8. He was taken for a laparoscopic appendectomy. Intraoperatively, the appendix could not be localized, so mobilization of the ileum and cecum from the lateral abdominal wall was done. As the ileocecal area was plastered to the abdominal wall, sharp and blunt dissection occurred. The ileocecal junction was not clearly visualized as there is omental adhesion in the area with redness at the terminal ileum with prominent wall capillaries. During dissection of the area to localize the appendix, an iatrogenic injury occurred at the cecum, and fecal matter was noticed coming out.

Surgery was converted to a midline laparotomy, opening the abdominal wall in layers. Full mobilization of the right colon laterally, sparing the right ureter and the duodenum the appendix still could not be localized. Therefore, the decision was taken to do a limited right hemicolectomy rather than a primary repair of the perforation to identify the presence of ileocecal pathology. The patient had an eventful recovery postoperatively and was discharged home in good health. Postoperative pathological examination of the right hemicolectomy specimen reported the absence of an appendix.

## Discussion

The vermiform appendix is a diverticulum of the caecum, which usually lies in the right lower quadrant of the abdomen. Due to asymmetrical growth of the lateral part of the caecum, the vermiform appendix is usually located medially, upwardly, and dorsally; in this position, it remains free after fixation of the caecum [4]. However, the appendix is believed to be the most variable abdominal organ because there are many possible configurations of its location. The range of positions of the tip of the appendix described in the literature are as follows: retrocecal (~38%), retrocolic (26%), subcaecal (14%), pelvic (8%), and preileal (3%) [5].

Morgagni identified congenital absence of the appendix in 1718, and it is an uncommon finding [6]. However, before making a diagnosis of agenesis or absence of the appendix, it is critical to understand that the vermiform appendix is a vestigial remnant that ranges in size from 2 to 20 cm, and, in some atypical cases, the appendicular tip may be found embedded inside the lumen of the caecum, a condition known as intussusception of vermiform appendicitis. The appendicular agenesis is presumed to be the result of intrauterine vascular accidents, auto amputations due to fibrous bands, and appendicular atresia [3]. One of the most common classifications of appendicular malformations is Collins’s classification. He classified appendicular malformations into 5 types: type 1 is absent of appendix and cecum; type 2 is rudimentary cecum and absent of appendix; type 3 is normal cecum without appendix; type 4 is normal cecum and rudimentary appendix; and type 5 is greatly enlarged and deformed cecum without appendix. In our study, we faced type 3, as there is a normal caecum and normal ileocecal junction but no appendix. This case was also reported by Arsenio *et al.*, but they found appendagitis that may happen due to torsion or spontaneous venous thrombosis of the involved epiploic appendage, and the operation was by open technique [7].

Acute appendicitis is one of the most common causes of abdominal pain and emergency surgery, with a mortality risk of 6–7% at its onset [8]. The diagnosis of acute appendicitis is based mainly on clinical observation using the Alvarado scoring system. This scoring system has a total score of 10; one point each is assigned to shifting abdominal pain in the right iliac fossa, loss of appetite (anorexia), nausea or vomiting, rebound tenderness, temperature 37.3C or more, and shift to the left (neutrophilia), and two points each are assigned for tenderness in the right iliac fossa and a white blood cell count of 10 000 mcL or more. An Alvarado score of 7 or higher carries 78% sensitivity and 100% specificity [9]. Based on this score, the majority of surgeons recommend appendectomy, especially in men, without any further investigation, but females may require ultrasonography for the exclusion of gynecological diseases. In the described case report, an Alvarado score of 8 urged and signaled a straightforward decision for appendectomy. However, a negative exploration revealed a limitation of the Alvarado scoring system. The most definitive treatment for acute appendicitis is appendectomy. In all cases of surgery for suspected appendicitis, if the appendix is found to be normal, the usual practice is to look for other sources of infection that could imitate symptoms such as those of acute appendicitis [10]. These include Meckle’s diverticulitis. Mesenteric lymphadenitis (more common in children) Salpingo-oophoritis, ovarian cyst and its complications, ruptured ectopic pregnancy, etc.

In cases where the appendix is not readily found, the whole caecum must be mobilized completely, the taenia coli are to be followed up to the junction where they meet, and the ileocolic region has to be carefully examined before the appendix is declared to be absent [10]. In our case, even with meticulous surgical exploration and search, the vermiform appendix was not found. The possibility of subserosal, subhepatic appendix, and Meckel’s diverticulum was ruled out, and interestingly, no mesenteric lymphadenopathy was observed perioperatively. A diagnosis of congenital absence of the appendix can be made once it is confirmed that there has been no previous abdominal surgery (including laparoscopy). If the diagnosis is seriously suspected, it is important to reassert this history postoperatively. All old hospital notes should be closely studied, and parental information should be sought on pediatric surgical procedures (if any). In the reported case, the final diagnosis of the absent appendix was established postoperatively when the pathology examination revealed an absent appendix in the right hemicolectomy specimen.

In conclusion, congenital agenesis, or absence of the vermiform appendix, is a very rare condition in the general population. Despite the rarity of this anomaly, we must consider it when we suspect acute appendicitis and with meticulous diagnosis and all appropriate means to avoid unnecessary surgery, which may lead to serious surgical complications. This case report mandates that the treating surgeons maintain a low threshold for considering nonappendiceal causes of abdominal pain, particularly in the presence of a significantly high Alvarado score. Imaging has its place in the diagnosis of atypical presentations of abdominal pain, particularly CT scans, and should be considered.

## Data Availability

Data will be made available on request.
